# Evaluating Circulating Biomarkers for Diagnosis, Prognosis, and Tumor Monitoring in Pediatric Sarcomas: Recent Advances and Future Directions

**DOI:** 10.3390/biom14101306

**Published:** 2024-10-16

**Authors:** Joaquín J. Maqueda, Alessandra De Feo, Katia Scotlandi

**Affiliations:** Laboratory of Experimental Oncology, IRCCS Istituto Ortopedico Rizzoli, 40136 Bologna, Italy; alessandra.defeo@ior.it (A.D.F.); katia.scotlandi@ior.it (K.S.)

**Keywords:** liquid biopsies, pediatric sarcomas, biomarkers, diagnosis, prognosis

## Abstract

Pediatric sarcomas present a significant challenge in oncology. There is an urgent need for improved therapeutic strategies for high-risk patients and better management of long-term side effects for those who survive the disease. Liquid biopsy is emerging as a promising tool to optimize treatment in these patients by offering non-invasive, repeatable assessments of disease status. Circulating biomarkers can provide valuable insights into tumor genetics and treatment response, potentially facilitating early diagnosis and dynamic disease monitoring. This review examines the potential of liquid biopsies, focusing on circulating biomarkers in the most common pediatric sarcomas, i.e., osteosarcoma, Ewing sarcoma, and rhabdomyosarcoma. We also highlight the current research efforts and the necessary advancements required before these technologies can be widely adopted in clinical practice.

## 1. Introduction

Pediatric sarcomas are developmental mesenchymal tumors, for which a strong demand for innovative treatments is cherished by patients, families, and physicians. They include a group of relatively rare malignancies with aggressive behavior and high tendency to metastasize and/or recur after treatment. Dose-intense chemotherapy and the evolving local treatments (surgery and/or radiation therapy) have led to a significant improvement in the chance of cure for the majority of patients with localized disease [1]. However, around half of the survivors suffer severe, disabling, or life-threatening conditions (i.e., risks of cardiomyopathy or secondary malignancies) [2].

For patients who do not respond to standard treatment, advances in therapy have stagnated, leaving critical gaps in care unresolved [3]. This underscores the pressing need for innovative approaches, including reliable biomarkers that can assess risk and response. Such biomarkers could help to stratify patients, enabling more targeted care and better resource allocation for high-risk individuals. Assessing new prognostic and predictive circulating biomarkers that reflect the biology/genetics of pediatric sarcomas may represent a promising tool to evaluate the status of the disease and potentially predict circulating disease, which is often responsible for tumor relapses [4].

Liquid biopsy can analyze biomarkers from various body fluids, mainly blood, saliva, and urine, to detect micro-metastases and evaluate tumor-derived indicators. While liquid biopsy techniques have been effectively explored in various cancer types [5], their application to aid in the treatment of sarcoma patients has remained behind, mainly due to the lack of common recurrent DNA mutations, which served as the foundation to liquid biopsy in many carcinomas [6]. Therefore, to advance liquid biopsy application for sarcomas, which have unique genetic and epigenetic profiles, innovative and integrated methods are still needed. This review explores recent advancements in liquid biopsy for the three most common pediatric sarcomas—osteosarcoma (OS), Ewing sarcoma (EWS) and rhabdomyosarcoma (RMS)—highlighting the opportunities to improve diagnosis, prognosis, and treatment decisions, while addressing the challenges of translating these assays into clinical practice.

## 2. Pediatric Sarcomas

Pediatric sarcomas are broadly classified into bone and soft-tissue sarcomas. Within these two umbrella categories, the most common sarcomas include OS and EWS among bone sarcomas, while RMS represents the most common soft-tissue sarcoma. However, this classification does not fully capture the genetic diversity of the different tumors. Genetically, sarcomas can be divided into those with well-defined molecular events and simple karyotypes, and those with complex genetic changes and unbalanced karyotypes [7,8]. Tumors with specific molecular alterations often exhibit small round cell histology, which does not exhibit a clear line of differentiation or indicates a potential differentiation block in a “stem” state. These tumors harbor disease-specific chromosome translocations, similar to those seen in hematologic malignancies [9]. The second group of sarcoma lacks consistent genetic alterations, although disruptions in the pRB and/or p53 regulatory pathways are frequently found, while *RAS* gene mutations are rare. Representatives of the two groups are EWS and OS, respectively [10].

EWS is characterized by the rearrangement of the *EWSR1* gene, which fuses with an ETS family transcription factor gene, most commonly *FLI1*. The resulting EWS::FLI1 fusion protein acts as an aberrant transcription factor, regulating gene transcription, RNA splicing, R-loop formation, and chromatin architecture [11]. The paucity of additional genetic alterations in the genome of EWS supports an indispensable role for the EWS::FLI1 fusion protein in tumorigenesis [12,13,14]. In addition, several studies have shown that not only the presence but also the levels of EWS::FLI1 expression can alter the epigenetic landscape of EWS, generating phenotypic heterogeneity with critical impact on tumor progression and treatment response (for a review, please refer to [15]). Unlike classic oncogenes, EWS::FLI requires precise expression for oncogenic activity: in permissive progenitor cells (i.e., mesenchymal or neural stem cells), excessive levels cause cell death, while insufficient levels fail to drive transformation. Within the tumorigenic window, high EWS::FLI1 expression increases proliferation and tumorigenic potential, whereas low levels enhance migration and metastatic capabilities [16,17,18], highlighting the need to precisely quantify the chimera besides revealing its presence.

On the other hand, OS has typically complex karyotypes with both numerical and structural chromosomal abnormalities that reflect significant genomic instability. Loss of pRB and p53 function or DNA helicases activity has been frequently associated with OS [19]. Additionally, approximately 25% of children or young adults with OS possess a highly penetrant germline mutation in a cancer-susceptibility gene [20]. OS is believed to originate from osteoprogenitor cells, with transformation potentially occurring at different stages of osteogenic differentiation and inducing disruptions of osteoblast terminal differentiation [21]. In general, OS is characterized by proliferation of malignant spindle cells with osteoblastic features and it is defined by the presence of uncalcified bone matrix (osteoid) [22]. From a genetic point of view, OS presents few recurrent single-nucleotide mutations but frequent, widespread large-scale cytogenetic abnormalities including multiple copy-number alterations, chromothripsis, and aneuploidy [23]. Recent multi-omics studies have led to identification of distinct subtypes characterized by peculiar molecular features [24,25,26], supporting the possibility of future precision medicine approaches.

RMS is the most frequent soft-tissue sarcoma in children. It derives from cells committed to muscle tissue differentiation [27]. Two major RMS subtypes are driven by fundamentally different molecular mechanisms. Alveolar RMS constitutes around 20% of RMS cases and is genetically characterized by chromosomal translocations involving the FOXO1 gene that fuses with PAX3 gene, in most of the cases (60%), or with PAX7 (20%) [28]. Conversely, embryonal RMS, the most prevalent subtype, exhibits aneuploid karyotypes with frequent chromosome gains or losses. Most embryonal RMS cases show loss of heterozygosity on chromosome 11, including the region 11p15.5, which encompasses genes encoding IGF2 and other growth factors, as well as tumor suppressors like CDKN1C [29]. Somatic driver mutations are also identified in up to 75% of ERMS, and frequently involve the RAS pathway, effectors of the PI3K pathway, genes controlling the cell cycle, and epigenetic modifiers [30]. While these genetic alterations have not yet shown prognostic value, their definition has led to a rapidly evolving classification system for RMS, with the WHO now recognizing four distinct RMS subtypes based on clinicopathological and molecular genetic features: embryonal, alveolar, spindle cell/sclerosing, and pleomorphic [31].

Despite the genetic, morphological, and biological differences among the three most common pediatric sarcomas, treatment strategies have historically been rather similar across these subtypes, comprising conventional chemotherapy, radiation, and surgery. However, recent advances in genome-wide and epigenome-wide profiling have revealed the significant molecular and genetic heterogeneity of sarcomas, leading to the identification of new reliable diagnostic and prognostic/predictive markers [32].

## 3. The Clinical Relevance of Liquid Biopsy in Pediatric Sarcomas

Tissue biopsy is the gold standard when establishing a diagnosis of sarcomas and has traditionally relied on histomorphological analysis combined with clinical and imaging features [1,33,34,35]. Molecular assays for fusion gene products and immunohistochemistry for overexpressed oncogenes have further enhanced diagnostic accuracy [36]. There are various types of biopsy techniques, including needle biopsy and open incisional biopsy [37]. However, they do not enable longitudinal monitoring of treatment response or early detection of relapse. Moreover, tissue biopsy cannot be repeated whenever it is necessary to characterize tumors during their spatial and temporal evolution, when they form metastasis, or change after treatments, all promises that are instead held by liquid biopsy [38].

Bodily fluids collection is quick, easy to repeat, and minimally invasive, features that are particularly desirable when patients are children, and their analysis provides sufficient sensitivity in detection of appropriate biomarkers for diagnosis and prognosis. Several circulating materials shed from tumors can be measured in liquid biopsies, such as circulating tumor cells (CTC), cell-free DNA (cfDNA) and RNA (cfRNA), and extracellular vesicle (EV)-associated cargo. This technology enables multiple parallel analyses, beneficial for monitoring tumors with high genetic and epigenetic heterogeneity but low mutation frequencies [39]. The aim of the next sections is to highlight the most relevant, recent advances involving CTCs, cfDNA/RNA, and EVs from the blood of patients with the three most common pediatric sarcomas.

### 3.1. Circulating Tumor Cells (CTCs)

CTCs are cancer cells that have detached from a primary tumor or metastatic deposits and entered the bloodstream [40]. These cells play a critical role in the metastatic process, as they can disseminate to distant sites and form secondary tumors [41]. Detecting and analyzing CTCs in whole blood may provide valuable insights into tumor progression and patient’s prognosis, as evidenced by the FDA-approved CELLSEARCH platform, which is intended for the enumeration of CTCs of epithelial origin [42]. Several clinical trials have been using this platform [43], which measures the epithelial cell adhesion molecule (EpCAM). Although EpCAM is commonly overexpressed in epithelial tumors, its expression in sarcomas is variable [44], yet it can still aid in detecting circulating sarcoma cells. Indeed, Tombolan et al. employed CELLSEARCH, in combination with desmin, a protein that is broadly expressed in RMS cells, to improve CTC identification in RMS patients’ blood and bone marrow samples [45]. They observed that metastatic RMS patients had a higher number of CTCs compared to those with localized disease. For selected cases, besides CTCs, the authors also molecularly profiled primary tumors and plasma cfDNA before and during treatment, finding several high-confidence somatic variants and demonstrating that both CTCs and cfDNA are circulating biomarkers that reflect the molecular makeup of primary RMS tumors.

As of now, the clinical relevance of CTCs as a prognostic or predictive marker in sarcoma remains unclear, primarily due to the small number of patients studied and the lack of specific markers expressed by sarcoma cells. Alternative methods for detecting and analyzing CTCs in sarcoma patients have been published and reviewed [46,47].

Because CTCs are frequently larger than that of normal circulating cells in blood, cell size has been used as a criterion for isolating sarcoma CTCs. Isolation by size of sarcoma cells (ISET) was first described by Chinen et al. [48]. Hayashi et al. [49] used a microfiltration system to demonstrate that CTCs in EWS, OS, and RMS could be readily detected at diagnosis. Their findings revealed that CTC levels decreased with effective treatment, yet they were still present in the blood of patients who showed no radiographic signs of disease before the onset of visible metastasis. After isolation, CTCs were characterized by immunocytochemistry, which confirmed the absence of white blood cell markers, by anti-CD45 (leukocyte common antigen) or anti-CD34 (hematopoietic and vascular-associated tissue marker), as well as the absence of epithelial-related markers, by anti-pan-cytokeratin (panCK) [48].

Another strategy for CTC detection in sarcomas is the use of common mesenchymal cell markers such as vimentin. Cell-surface vimentin (CSV) emerged as a potential general marker for sarcomas, including OS and EWS [50]. The presence of CSV-positive CTCs in patients with sarcoma was associated with poorer overall survival [51], and metastatic patients had a higher CTC number, suggesting that CTCs may have prognostic value [52]. In addition, recent research has demonstrated that protein–antibody combinations using GD2 or cytokeratins and CSV are more effective in capturing CTCs from various sarcoma types [52,53].

In the few sarcomas characterized by the expression of specific makers, such as CD99 in EWS or PAX3 in RMS, CTCs could be detected by flow cytometry [54]. In particular, flow cytometry was used to identify EWS circulating cells by exploiting the common expression at high levels of CD99 and the absence of CD45 expression to distinguish EWS cells from other cell types [55]. Benini et al. [56] reported the possibility to isolate EWS CTCs in peripheral blood using immunomagnetic separation with microbeads and CD99 monoclonal antibody. To confirm the cancerous origin, isolated cells were subjected to molecular analysis to detect specific fusion transcripts, such as EWS::FLI1.

Several studies in OS have shown a strong correlation between CTCs count and tumor stage or prognosis. As an example, Dai et al. [57] found that CTCs were detected in the majority of OS patients and that CTC count correlated with staging and tumor size throughout the whole course of treatment. Particularly, CTCs that were found positive for the expression level of IMP3, an RNA-binding protein reported to act as an oncogene in several tumors including sarcomas (for a review, see Mancarella et al. [58]), were found to be related to the metastasis formation. Another study demonstrated that the expression of survivin, an apoptosis inhibitor, in OS CTCs is associated with worse disease prognosis of OS [59]. Several studies have shown that variations in CTC counts following therapy or surgical resection can indicate the tumor’s sensitivity to treatment and may serve as a reliable indicator of metastasis [60,61,62,63].

In general, these studies showed the potential of CTC markers in sarcomas (summarized in Table 1) but large clinical trials are needed to improve the real contribution of CTCs to the understanding of tumor progression. CTCs have the great advantage that they can be molecularly characterized and used in functional assays, but they are very rare in peripheral blood and the specificity of CTC markers needs to be improved and validated.

### 3.2. Cell-Free DNA (cfDNA)

Cell-free DNA (cfDNA) is released into bodily fluids through processes such as apoptosis or necrosis and is typically found as degraded fragments [64]. Tumor-derived cfDNA (ctDNA) is shed from tumor cells and represents the entire tumor genome. Therefore, ctDNA analysis, mainly from blood [65], provides a comprehensive view of the tumor’s overall genetic landscape, revealing heterogeneity that may be missed with tissue biopsies [66]. Total ctDNA may contain genetic alterations in patients with cancer and have been shown to have diagnostic and prognostic potential. Several FDA-approved cfDNA tests are clinically used in cancer, such as FoundationOne Liquid CDx, which analyzes cfDNA to detect genetic alterations for solid tumors [67]; cobas EGFR mutation test V2 for non-small-cell lung cancer [68]; Guardant360 CDx that detects actionable mutations in lung and breast cancer [69]; Epi proColon 2.0, which looks for methylated Septin9 DNA in colorectal cancer [70]; and Therascreen PIK3CA RGQ PCR, which detects actionable alterations in breast cancer [71]. While these platforms are well-established for other cancers, their application in sarcomas is still under exploration. Research has shown that cfDNA analysis can be particularly useful in pediatric cancers with low mutational burden, such as Ewing sarcoma and other pediatric sarcomas [72].

In healthy individuals, cfDNA is typically present in plasma at low concentrations, compared to cancer patients [73]. However, this level can rise in response to conditions that induce tissue stress [74,75]. It has also been shown that higher concentrations of ctDNA at initial diagnosis could be used as a prognostic biomarker of inferior outcomes in EWS [76]. Shulman and colleagues used two next-generation sequencing (NGS) methods, NGS hybrid capture and ultra-low-pass whole-genome sequencing (ULP-WGS), to detect ctDNA in blood from EWS patients and OS patients with localized disease. ctDNA was detected in newly diagnosed patients, and in 13.8% of EWS patients, ctDNA contained an EWSR1 translocation. Although the presence of sarcoma-derived cfDNA did not significantly correlate with patient outcomes in this study, detectable ctDNA at diagnosis was linked to higher mortality rates [77].

Udomruk et al. analyzed the size and mutation profiles of cfDNA in blood of pre-treatment OS patients vs. healthy donors. They found that size of cfDNA was significantly shorter in metastatic OS patients, it was related to poorer survival, and that shorter cfDNA was a major source of mutations, with a 2.3-fold increase of copy-number variants (CNV). In addition, the levels of cfDNA were substantially increased in OS patients, mainly in those with advanced OS, supporting the use of cfDNA size, levels, and mutations for clinical applications [78].

WGS has also been applied to find CNVs in paired cfDNA and tissue DNA samples from pediatric patients’ cohorts. Overall, the authors observed a good concordance between CNVs in tissue DNA and cfDNA, being low-quality samples the main cause of discordances [79]. Similarly, Barris et al. focused on seven genes commonly mutated in OS, including TP53 and RB1. Specific somatic mutations that were identified in the primary OS tumors were also detected in the ctDNA during periods of clinical relapse, indicating that ctDNA could be a potential value for monitoring disease progression and recurrence [80].

Moreover, the potential of cfDNA in pediatric sarcomas was demonstrated by a recent study that analyzed cfDNA from 95 EWS patients and 31 with other pediatric sarcomas, including OS and RMS, compared to healthy controls [72]. Researchers developed a novel approach combining three methods to enhance the detection of ctDNA in EWS: CNV quantification based on read depth and EWS::ETS quantification by WGS and by digital droplet PCR (ddPCR). Then, they focused on global DNA fragmentation, and they found that shorter cfDNA were enriched in cfDNA samples with high ctDNA levels. Using this finding, they proposed using a CNV profile of shorter cfDNA (ctDNA-related) to stratify groups of patients, across disease progression, with high subclonal resolution. This study also introduced the LIQUORICE algorithm, a bioinformatics tool for identifying ctDNA based on fragmentation patterns that reflect the chromatin structure of the primary tumor. In summary, they created machine learning classifiers that utilize fragment-based techniques to accurately differentiate between patients with EWS and healthy individuals, as well as between EWS patients and those with other sarcomas. This approach establishes a liquid biopsy method that operates independently of recurrent genetic aberrations.

Another key application of ctDNA from blood plasma is to monitor minimal residual disease (MRD) in EWS and OS patients using ddPCR or NGS [81,82]. By employing ddPCR, researchers can detect patient-specific fusion gene breakpoints, such as those involving the EWS::FLI1 fusion. This method allows for real-time monitoring of disease progression and response to treatment, offering a less invasive and more frequent alternative to traditional biopsies and imaging techniques [81]. ddPCR was also applied to detect ctDNA in patients with OS by methylation-based biomarkers. These CpG methylation markers were highly specific for OS and correlated with disease progression when samples from patients before and after surgical resection were analyzed. Additionally, cfDNA correlated with survival, highlighting the use of cfDNA to stratify patients [83].

In RMS, the presence of ctDNA has been associated with tumor burden throughout treatment in several studies [84,85]. ctDNA can be analyzed using various methods that focus on genetic changes specific to RMS. For instance, the fusion PAX3/7::FOXO1 was detected in cfDNA of a diagnosed alveolar RMS patient [84]. Gallego et al. (2006) also demonstrated the detection of RMS-specific fusion genes, including PAX3::FOXO1, using reverse transcriptase-polymerase chain reaction (RT-PCR) methods on cfDNA, highlighting the potential for early detection and monitoring of disease [86]. Moreover, some studies demonstrated that the unique methylation profiles in RMS can be detected in ctDNA from plasma samples at diagnosis, using cell-free reduced representation bisulfite sequencing (cfRRBS) to accurately classify the tumor as either embryonal or alveolar [87], or to predict poor prognosis by detecting RASSF1A-M (silenced by methylation in several tumors) positive patients [88]. CNAs have been analyzed in RMS by shallow WGS (shWGS). Recently, Van Paemel et al. [87] showed that shWGS data from cfDNA can complement CNA analysis performed on the primary tumor. Additionally, Abbou et al. reported that ULP-WGS and Rhabdo-Seq effectively detected ctDNA in RMS patients, associating detectable ctDNA with significantly worse event-free and overall survival, and reinforcing ctDNA potential as a prognostic marker in RMS [89].

In summary, by analyzing cfDNA, clinicians can gain valuable information about tumor genetic alterations, response to treatment, and tumor progression (Table 2). As technologies and methodologies continue to advance, the integration of ctDNA analysis into routine clinical practice may hold the promise of enabling more precise and timely interventions that are tailored to the evolving characteristics of each patient’s subtype.

### 3.3. Cell-Free RNA (cfRNA)

cfRNA, including messenger RNA (mRNA) and non-coding RNAs such as microRNAs (miRNAs), can be found in bodily fluids like blood [90,91]. These RNAs can provide valuable insights into the molecular landscape of various diseases, including pediatric sarcomas [92]. miRNAs, the most abundant cfRNA molecule in blood [93,94], are small non-coding RNA molecules that regulate gene expression by inhibiting their target mRNAs [95]. They are especially interesting as biomarkers due to their stability in circulation [96], resistance to degradation [97], and ability to reflect the pathophysiological state of their tissue of origin [98,99]. miRNAs can exist freely in the bloodstream or within EVs, which protect them from enzymatic degradation and facilitate their transport to distant cells, influencing various biological processes [100]. In cancer, miRNAs play crucial roles in cell apoptosis, invasion, metastasis, and disease progression. They have valuable applications in diagnosis, prognosis, and therapy [101]. In bone tumors, miRNAs epigenetically regulate osteogenesis [102,103] and osteoclast differentiation, while in RMS they play a significant role in myogenic differentiation [104].

#### Circulating miRNA

Various studies have identified specific miRNAs or miRNA panels in blood as diagnostic or prognostic biomarkers that can distinguish between OS patients and control groups (reviewed in Huber et al. 2023 [105]). Notably, one of the studies investigated serum miRNA profiles of >1000 patients with bone and soft-tissue tumors representing more than 43 histological subtypes. They highlighted how the expression level of seven serum miRNAs (miR-4736, miR-6836-3p, miR-4281, miR-762, miR-658, miR-4649-5p, and miR-4665-3p) distinguished sarcoma patients from patients with benign lesions or from healthy controls, indicating that serum miRNA profiles can diagnose sarcoma accurately [106]. In another study, miR-199a-5p concentrations in serum were significantly higher in pre-operative OS patients than in controls, and then significantly decreased after surgery [107]. In addition, miR-21 and miR-221 have been shown to be upregulated in OS and other solid tumors, and related to tumor processes, either in circulating or in tissue samples [108,109,110,111,112]. miR-21 may be used as a predictive biomarker to identify patients who are likely to respond poorly to chemotherapy at diagnosis, and miR-221 was proposed as a promising prognostic biomarker in OS [113].

Kosela-Paterczyk et al. explored the signatures of miRNAs in the serum of sarcoma patients, finding 24 and 42 miRNAs that differentiated healthy controls from OS and EWS patients, respectively. Of these, two single miRNAs (miR-142-3p and miR-9-3p) showed excellent discriminatory power, between EWS and healthy controls, and the authors proposed two different miRNA signatures as diagnostic classifiers, for OS: miR-133a, miR-223-3p, miR-450b-5p, and miR-548q; for EWS: miR-424-5p, miR-3173-3p, miR-142-3p, and miR-4746-5p [92]. In EWS, circulating levels of miR-34a, which is transcriptionally induced by p53 and acts as an oncosuppressor, was lower in metastatic patients compared to those with localized tumors [114]. Consistently, a decrease in miR-34a serum levels was associated with distant metastasis in OS [115,116]. Similar evidence was reported for miR-125b, whose lower expression predicted poor response, and was found to be associated with advanced stages in EWS or OS [117,118].

In RMS patients, circulating miR-26a and miR-30b/c levels were found to be reduced compared to controls and low miR-26a plasma levels were associated with adverse outcomes [91]. Conversely, high serum miR-26a-5p levels in OS were linked to a poorer survival than those with low miR-26a-5p levels, suggesting that miRNA expression can be highly specific to particular tumor types [119]. In addition, muscle-specific miRNAs were found to be elevated in sera of patients with RMS while the oncosuppressor miR-206 was shown as the best biomarker for RMS prediction [120]. A decrease of this miRNA in serum was also linked to tumorigenesis and tumor progression in OS [121].

EVs are lipid-bilayer membrane-delimited particles released by all cell types [122] that include exosomes, microvesicles, and apoptotic bodies, each differing in size, biogenesis, and cargo composition [123]. Discriminating between these EV subtypes and their function is challenging due to their overlapping characteristics [124], the complexities related to the isolation of a single subtype, and the lack of demonstration of subcellular origin [122]. In this review, we will consider all subtypes under the term “EVs”.

EVs fulfill multiple roles in cell-to-cell communication by carrying proteins, lipids, and nucleic acids, such as mRNA and miRNA, which can influence critical functions in cancer [125,126,127]. Analysis of EV cargo in bodily fluids has been extensively performed to gain insights into disease status and treatment responses [128,129]. Regarding clinical application in cancer prediction, the first and only commercially available EV-based test, the ExoDx™ Prostate (IntelliScore) [130], utilizes urinary EV-RNA transcripts and has reduced the need for tissue biopsies by 27% in prostate cancer patients [131]. Encapsulated in EVs, cfRNA is protected from degradation by extracellular enzymes, maintaining their stability and functional integrity. This protection enhances their potential as reliable biomarkers in liquid biopsies [132]. There are several recent clinical trials investigating EV-miRNAs in sarcomas (NCT03800121, NCT03108677, and NCT03895216) and there is increasing evidence that EV-miRNAs could be used as circulating biomarkers.

Several studies have shown the relationship between EV-miRNA and metastasis in OS. In particular, Gong and colleagues isolated EVs from both patients’ serum and metastatic OS cell lines, identifying miR-675 as a potential biomarker for OS metastasis due to its biological role in experimental models and upregulation in the serum of patients [133]. Other studies found that the EV-derived miR-101 could inhibit metastasis, as its expression was found to be significantly lower in OS patients compared to healthy controls, with even lower levels in patients with metastatic disease, and it was associated with worse overall survival [134]. EV-miRNA profiling led to the finding of diverse miRNA expression between OS patients and healthy controls. miR-92a-3p, miR-130a-3p, miR-195-3p, miR-335-5p, and let-7i-3p were upregulated in OS patients and functional studies indicated that miR-195-3p regulates OS cell proliferation and invasion [135]. Furthermore, a distinctive profile of several EV-miRNAs, including miR-124, miR-133a, miR-135b, miR-148a, miR-199a-3p, miR-27a, miR-385, and miR-9, has been proposed to differentiate chemotherapeutic responses in OS patients [136].

In EWS, a study identified 62 differentially expressed EV-derived miRNA candidates in vitro and validated a signature of 46 of these miRNAs in the sera of patients, which accurately distinguished pathology-confirmed EWS cases [137].

High levels of miR-1246 in serum-derived EVs from RMS patients were found to potentially disrupt the Wnt/β-catenin signaling pathway by targeting GSK3β in recipient cells [138]. In alveolar RMS, the PAX3-FOXO1 oncogene alters EV-miRNA content, particularly increasing miR-486 levels in EVs, which acts as an effector of PAX3-FOXO1, and promotes cell migration, invasion, and colony formation in recipient cells [139].

In general, several studies indicated a potential role of circulating miRNAs and EV-encapsulated miRNAs as biomarkers of diagnosis, prognosis, and treatment response (Table 3 and Table 4), but data are very heterogeneous and not validated in large, prospective clinical studies. This limits their use in clinical application.

**Figure 1:**
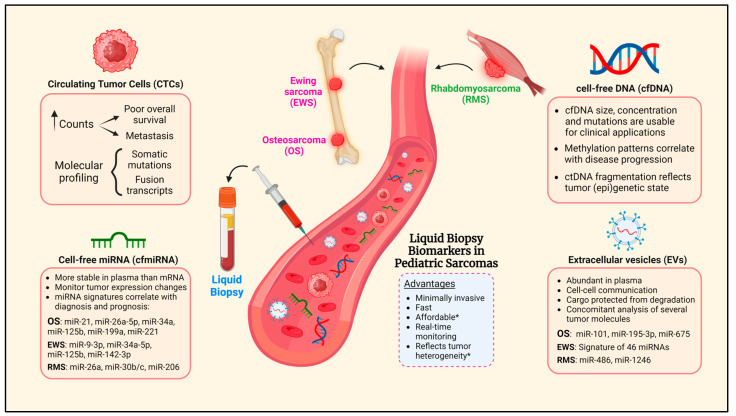
Overview of liquid biopsy biomarkers and their role in pediatric sarcomas. Features and clinical relevance of CTCs, cfDNA, cfmiRNA, and EVs. Proportions and concentrations of CTCs, cfDNA, miRNAs, and EVs shown in the figure are illustrative and not intended to reflect their actual levels in the bloodstream. Red blood cells represent hematopoietic cells in blood, which are not the focus of this review. * May not be verifiable in all circumstances.

Recent findings have highlighted the potential of liquid biopsies to enhance diagnosis and treatment in pediatric sarcomas. In this review, we have discussed the roles of various liquid biopsy biomarkers, including CTCs, cfDNA, circulating miRNAs, and EV-miRNAs. Figure 1 summarizes these biomarkers and their clinical relevance in pediatric sarcomas.

## 4. Challenges and Future Perspectives

Liquid biopsies offer a wide range of potential clinical applications, such as early cancer detection, prognosis, monitoring response, identification of potentially actionable alterations, detection of resistance mechanisms, assessing tumor heterogeneity, and personalization of treatments [140]. However, their clinical utility still requires validation, as current limitations include assay reproducibility and sensitivity. These can be improved through standard operating procedures and reference materials to control pre-analytical factors [141,142]. For instance, profiling CTCs, ctDNA, ctRNA, or EVs-associated miRNA has emerged as a promising tool for biopsy-free tumor genotyping. However, so far, the low abundance of CTCs, the scarcity of ctDNA, the short half-life of RNA [143], and the high variability in size, content, and origin of EVs have substantially limited the clinical utility of these approaches [141].

From a biological perspective, it is still unclear whether liquid biopsy accurately represents the genomic diversity within tumors [144], which is particularly relevant for sarcomas due to their high level of genetic and epigenetic heterogeneity. This point could be clarified, for example, by implementing the comparative studies with single-cell genomic profiling of tumor tissues.

Most circulating material in blood are released by blood or endothelial cells, not diseased tissues like tumors, making it difficult to distinguish tumor cells from normal hematopoietic cells in the blood [145]. In addition, while tumors do release cfDNA and miRNA, tumor-specific nucleic acids are often a small fraction compared to normal cell-derived material [146], influenced by tumor size and expression levels.

To tackle these challenges, screening for multiple cancer-associated mutations can improve ctDNA detection, while combining data such as DNA methylation and mutation status enhances the specificity of the analysis. Although circulating miRNAs are sensitive to physiological factors, EV-derived miRNAs are more stable, tissue-specific, and protected from degradation [147]. Advances in isolation methods based on surface markers of EVs offer the potential for better differentiation of disease-specific miRNAs from those produced physiologically. However, most research on mRNA and miRNA in blood remains preliminary, and clinical validation with standardized protocols is necessary to establish their diagnostic and prognostic value [122,148,149].

In spite of these challenges, the future perspectives for EV-RNAs in sarcoma management are promising. The FDA approval of ExoDx™ for prostate cancer shows the feasibility of liquid biopsy biomarkers in clinical practice. Technological advances, like high-throughput sequencing and digital PCR, are improving miRNA detection sensitivity, while nanotechnology is enhancing EV isolation and miRNA delivery for therapeutics. A deeper understanding of EV-miRNA biogenesis will refine biomarker and therapeutic target identification.

Large, longitudinal clinical studies using standardized methods are needed for advancing liquid biopsy research, especially for rare pediatric sarcomas, where clinical trials suffer from small patient cohorts [65]. Furthermore, given the difficulty in obtaining samples from these rare tumors, current research is predominantly based on retrospective studies with considerable variability in sample collection, processing, and analysis. Expanding cohorts, harmonizing protocols, and standardizing procedures are crucial to improving the reliability of liquid biopsies in these rare cancers.

## 5. Conclusions and Future Perspectives

Being non-invasive and suitable for repeated analysis of molecular biomarkers, the application of liquid biopsy offers major potential advantages for pediatric sarcomas. While current limitations remain, ongoing improvements in detection technologies, standardization, and sample collection will enhance their clinical applicability. As this field evolves, liquid biopsy is expected to refine diagnosis and influence clinical study design. Ethical considerations, particularly regarding patient communication, will be important as liquid biopsy becomes more integrated into sarcoma management. Ensuring patients are informed and engaged in the decision-making process will be critical for liquid biopsy successful implementation in clinics.

## Figures and Tables

**Table 1:** Markers for the detection and isolation of circulating tumor cells (CTCs) in the most common pediatric sarcomas.

Marker	Description	Cancer Type	References
EpCAM	Epithelial tumor marker. Variable expression in sarcomas. High levels at diagnosis correlated with reduced RMS patients’ overall survival.	Epithelial tumors, Sarcomas	[44,57,59]
Desmin	Mesenchymal marker, abundantly expressed in RMS. Used in combination with EpCAM, CTC detection remarkably increased.	RMS	[44,45]
CD45	Leukocyte common antigen. Absence of CD45 is used to characterize CTCs.	Sarcomas	[44,45,48,49,50,52,55,57,59]
CD34	Hematopoietic and vascular-associated tissue marker. Absence is used in CTC characterization.	Sarcomas	[48,55]
PanCK	Epithelial-related markers. Absence is used in CTC characterization in non-epithelial cancers such as sarcomas.	Sarcomas	[44,45,48,52,57]
Vimentin	Common mesenchymal marker. CSV is a general marker for sarcomas and is associated with poorer survival.	Sarcomas	[48,49,50,51,52,57]
GD2	Ganglioside, highly expressed in OS cells. In combination with CSV, the capture efficacy of OS cells was significantly increased.	OS	[52,53]
CD99	Membrane marker related to EWS malignancy. Used in CTC detection in combination with CD45.	EWS	[55,56]
EWSR1::FLI1	Specific EWS fusion chimera. Used in combination with CD99 to detect EWS-derived CTCs.	EWS	[56]
IMP3	RNA-binding protein, acts as an oncogene. Expression in CTCs correlates with metastasis formation in OS.	OS	[57]
Twist	Mesenchymal marker.	OS	[57,59]
Survivin	Apoptosis inhibitor. Expression in CTCs associated with worse prognosis in OS.	OS	[59]

**Table 2:** Overview of ctDNA features and detection methods in the most common pediatric sarcomas: technologies, findings and clinical implications.

Indicators	Method	Sarcoma Type	Key Findings/ Clinical Implications	References
cfDNA levels	ddPCR NanoDrop NGS hybrid capture ULP-WGS	EWS OS	Higher levels found in advanced OS and EWS, correlated with tumor burden and progression, and predictive of patient outcomes.	[76,77,78,82,83,85]
cfDNA fragment length	Electrophoresis WGS	EWS OS	Shorter cfDNA associated with poorer surviv68al in metastatic OS patients and identified as a major source of mutations and increased CNV.	[72,78]
cfDNA fragmentation patterns	WGS LIQUORICE algorithm	EWS OS RMS	Non-genetic way of detecting tumor-derived DNA in the cfDNA samples. Prognostic biomarker in EWS. Reflects the characteristic chromatin structure in primary EWS tumors and may be associated with prognosis.	[72]
Fusion gene detection (EWS::ETS, PAX3/7::FOXO1)	WGS ddPCR RT-PCR	EWS OS RMS	Detects specific fusion gene breakpoints in ctDNA; enables real-time monitoring of disease progression and MRD and response to treatment.	[72,81,84,86]
Genomic alterations	NGS (sh)WGS ULP-WGS RRBS Rhabdo-seq	EWS OS RMS	Somatic mutations and CNVs can be found in cfDNA and show good concordance with tissue DNA, enabling the tracking of disease progression in individual patients.	[72,78,80,85,87,89]
DNA Methylation Markers	RRBS ddPCR shWGS	EWS OS RMS	Correlation with disease progression and survival. Methylation profiles help classify tumor subtypes and predict prognosis.	[72,83,87,88]

**Table 3:** Circulating miRNA biomarkers in the most common pediatric sarcomas. We have omitted certain miRNAs that were already covered in detail in a previous review [105], focusing instead on new or key findings in EWS, OS, and RMS.

miRNA	Description	Cancer Type	Source	References
miR-4736 miR-6836-3p miR-4281 miR-762 miR-658 miR-4649-5p miR-4665-3p	Diagnostic signature. Seven miRNA expressions distinguished sarcoma patients from patients with benign lesions or from healthy controls.	Sarcomas	Serum	[106]
miR-199a-5p	Biomarker for cancer detection and monitoring, showing higher expression in pre-operative patients compared to healthy controls, with a notable decrease post-surgery.	OS	Serum	[107]
miR-21	Diagnostic and prognostic biomarker proposed to identify patients who are likely to respond poorly to chemotherapy at diagnosis. Expression levels correlated with metastasis status and histological subtype of the patients.	OS	Serum/ Plasma	[108,109,113]
miR-221	Upregulated in patients’ blood, with higher levels observed in those at advanced stages. Diagnostic and prognostic biomarker.	OS	Serum/ Plasma	[110,113]
miR-142-3p miR-9-3p	Single miRNAs with high discriminatory power between patients and healthy controls. Diagnostic biomarkers.	EWS	Serum	[92]
miR-133a miR-223-3p miR-450b-5p miR-548q	Signature with high discriminatory power. Multi-miRNA diagnostic classifier.	OS	Serum	[92]
miR-424-5p miR-3173-3p miR-142-3p miR-4746-5p	Signature with high discriminatory power. Multi-miRNA diagnostic classifier.	EWS	Serum	[92]
miR-34a(-5p)	High levels inversely correlate with tumor volume, metastasis, and chemotherapy resistance. Low levels indicated poor clinical outcomes. It could distinguish patients from controls. Biomarker for diagnosis, disease progression, and therapy response.	EWS OS	Serum/ Plasma	[114,115,116]
miR-125b	Expression levels decreased in patients when compared with healthy controls. Significantly downregulated in poor responders. Biomarker for diagnosis, prognosis, and prediction of treatment response.	EWS OS	Serum/ Plasma	[117,118]
miR-26a(-5p)	Diagnostic/prognostic biomarker. Higher levels in OS patients. Associated with fusion status and adverse outcome in RMS.	OS RMS	Serum/ Plasma	[91,119]
miR-30b/c	Prognostic biomarker. Levels were found to be reduced in patients compared to controls.	RMS	Plasma	[91]
miR-206	Diagnostic/prognostic biomarker. Expression levels significantly increased in patients and could differentiate them from healthy individuals.	OS RMS	Serum/ Plasma	[91,120,121]

**Table 4:** EV-miRNA biomarkers in the most common pediatric sarcomas.

EV-miRNAs	Description	Cancer Type	Source	References
miR-675	Expression is higher in metastatic OS patients-derived exosomes. Potential prognostic biomarker.	OS	Serum	[133]
miR-101	Potential diagnostic biomarker. Expression levels were reduced in EVs from patients compared with healthy controls and even lower in patients with metastasis.	OS	Plasma	[134]
miR-92a-3p miR-130a-3p miR-195-3p miR-335-5p let-7i-3p	Expression levels were upregulated in patients vs. healthy subjects. Potential targets for treatments and biomarkers for diagnosis.	OS	Serum	[135]
miR-124 miR-133a miR-199a-3p miR-385	Validated diagnostic biomarkers that can predict response to chemotherapy. Expression levels were reduced in poor responders.	OS	Serum	[136]
miR-135b miR-148a miR-27a miR-9	Biomarkers to predict response to chemotherapy. Significantly overexpressed in poor responders.	OS	Serum	[136]
Signature of 46 miRs	miRNA classifiers of EWS family of tumors. Biomarkers for diagnosis and monitoring.	EWS	Plasma	[137]
miR-1246	High levels in patient’s serum. Potential diagnostic biomarker and promoter of oncogenesis.	RMS	Serum	[138]
miR-486	Enriched in serum–derived EVs from patients. Very high levels in the one patient with fusion-positive alveolar RMS. Decreased after chemotherapy and in cancer remission. Potential role in diagnosis, assessment of response and follow-up of patients after treatment	RMS	Serum	[139]

## Data Availability

Being a review, no new data were created and analyzed in this study.
